# Inter-atrial Septum Stenting in Congenital Heart Disease Patient: A Case Series in Indonesia

**DOI:** 10.2174/011871529X320825240925073605

**Published:** 2024-10-15

**Authors:** Radityo Prakoso, Yovi Kurniawati, Sisca Natalia Siagian, Aditya Agita Sembiring, Damba Dwisepto Aulia Sakti, Brian Mendel, Olfi Lelya, Oktavia Lilyasari

**Affiliations:** 1 Division of Pediatric Cardiology and Congenital Heart Disease, Department of Cardiology and Vascular Medicine, National Cardiovascular Centre Harapan Kita, Universitas Indonesia, Jakarta, Indonesia;; 2 Department of Cardiology and Vascular Medicine, Sultan Sulaiman Government Hospital, Serdang Bedagai, North Sumatera, Indonesia

**Keywords:** Congenital heart disease, developing country, duct-dependent, high-risk, inter-atrial septum stenting, low-frequency, single ventricle

## Abstract

**Background:**

Inter-atrial septum (IAS) stenting in duct-dependent congenital heart disease patient has shown to be an effective way to maintain inter-atrial blood flow, however it is still considered a high risk procedure and inter-atrial septum stenting remains a low-frequency procedure.

**Methods:**

A single-center observational cohort study was carried out at the National Cardiovascular Center Harapan Kita (NCCHK) between April 2019 and April 2023. This study included duct-dependent congenital heart disease patients. The extracted data were baseline characteristics, clinical findings, complications, and outcomes of the patients.

**Results:**

Eleven patients with duct-dependent physiology were intervened with inter-atrial septum stenting. The patients were 4 females and 7 males with the median age of implantation being 150 days (range 11-703 days) and the median weight being 3.9 (range 2.8-9) kg, with 2 patients weighing less than 3 kg. The average stent diameter was 8.50 (2.03) mm with an average length of 24.45 (7.94) mm. Non-restrictive atrial flow was successfully achieved in 90.90% of the procedures, corresponding to 10 patients.

**Conclusion:**

Inter-atrial septum stenting in duct-dependent congenital heart disease patients produces reliable patency with a very good intra-procedural success rate.

## 1 INTRODUCTION

Atrial septal patency must be maintained in patients with duct-dependent physiology in order to maintain hemodynamic stability. A constricted inter-atrial septum is typically addressed by performing a traditional septostomy (Rashkind) within the first few days or weeks of infancy. This procedure is generally sufficient until corrective or palliative surgery can be undertaken. In the context of a thick atrial septum, the implantation of an inter-atrial septum (IAS) stenting is recommended due to its controlled, predictable, and long-lasting atrial communication [1-3].

Yet, due to its high risk, inter-atrial septum stenting remained a low-frequency procedure in certain congenital heart disease and idiopathic pulmonary arterial hypertension patients [4, 5]. Therefore, the goal of our study is to describe our experience with the outcomes of patients who underwent inter-atrial septum stenting.

## 2 METHODS

### 2.1 Study Design and Settings

Eleven patients with duct-dependent physiology were admitted to our center between April 2019 and October 2023 for this retrospective, single-center observational cohort study. This study received ethical approval from the Institutional Review Board of the National Cardiovascular Center Harapan Kita (Approval No. LB.02.01/VII/018/KEP018/2023) and was conducted in accordance with the Helsinki Declaration of 1975. All patients utilizing emergency, outpatient, and inpatient services are documented within the hospital's electronic medical record (EMR) system. Participants who met the inclusion criteria were subsequently followed up on and evaluated while in the hospital. Informed consent has been obtained from each subject for this study.

### 2.2 Interventional Procedure

Under general anesthesia, all procedures were conducted with fluoroscopic guidance. The indication of IAS stenting is: (1) relief of left atrial hypertension, (2) maintenance of systemic cardiac output, (3) inter-circulatory mixing, (4) reducing pulmonary vascular resistance of biventricular circulation, or (5) reducing pulmonary vascular resistance in univentricular circulation. A 6-9 Fr sheath was inserted into a femoral vein for the transcatheter approach. For stability, a guidewire was positioned in a left-sided pulmonary vein, curved in the left atrium, or passed over the left atrioventricular valve and through the ventricle to the great artery emerging from the ventricle after crossing the atrial septum. If the atrial septum was intact, a Brockenbrough needle was used to perforate it, which was then replaced with a guidewire as previously described. Following pressure readings and angiography, a long sheath was advanced directly into the left atrium, and the stent was successfully implanted.

The size of the atrial septal stent is determined based on hemodynamic indications. In cases of univentricular hearts with mitral stenosis, a very large atrial stent is necessary to fully relieve left atrial hypertension, thereby optimizing hemodynamics for a bidirectional Glenn shunt. In such scenarios, the goal of atrial septal stenting should be to achieve a stent diameter ranging from 8 to 12 mm. However, the anatomical constraints of the left atrium in very small infants with hypoplastic left heart syndrome limit the stent size to 4-6 mm. Furthermore, complete relief of left atrial pressure in these neonates leads to a significant decrease in pulmonary vascular resistance and pulmonary artery pressures. The pressures in the aorta perfused by the arterial duct will decrease once the pressures in the pulmonary arteries do. Therefore, it is preferable to have a somewhat restricted atrial communication where the left atrial pressure is 4-6 mmHg higher than the right atrium [2].

The stent/balloon assembly was typically deployed by passing it to the end of the sheath in the left atrium. Subsequently, the sheath was withdrawn, revealing the distal half of the stent, which was then inflated within the left atrium. The partially inflated stent and sheath were then repositioned to engage the inter-atrial septum. Following this, the proximal portion of the stent was exposed in the right atrium and fully dilated. Alternatively, in other procedures, the stent was delivered to the inter-atrial septum through the long sheath, the sheath was then removed entirely, and the balloon was fully inflated in a single maneuver. Post-dilation flow was assessed using fluoroscopy and echocardiography, and if flow remained partially restricted or if the stent showed no waist, a larger balloon was employed. Following the procedure, all patients received aspirin-based antiplatelet medication until the stent was removed.

The size of the stent in duct-dependent pulmonary circulation who received PDA stenting should resemble the conventional modified-Blalock-Thomas-Taussig shunt (mBTTs) size. However, The size of the stent should be as large as possible to simulate systemic circulation in duct-dependent systemic circulation. Neither cardiopulmonary bypass nor ECMO were employed throughout the treatment, and the patient's normothermia was maintained.

### 2.3 Follow-up and Outcomes

The individuals’ information was collected, which included their age, gender, birth weight, weight at stent placement, type of CHD, procedural risk for major adverse events, stent condition during last follow-up echocardiography, perioperative/ 30-day mortality, intra-, and post-procedural complication. The individuals were observed until they were released from the medical facility. During hospitalization, clinical findings such as the severity of duct-dependent physiology were documented. If the stent was in place with unrestricted interatrial flow immediately after the procedure, it was considered a successful implantation. Clinical success was determined as the effective placement of the stent and discharge from the hospital without requiring additional intervention.

## 3 RESULTS

### 3.1 Patients Characteristic

This study included 11 patients with duct-dependent physiology. Table **1** summarizes the initial traits of the patients. Among them, there were 4 females and 7 males, with a median age at implantation of 150 days (ranging from 11 to 703 days) and a median weight of 3.9 kg (ranging from 2.8 to 9.0 kg), including 2 patients weighing 3 kg or less. All patients exhibited single ventricle physiology and had notably high pre-procedural risk scores according to the Catheterization RISk Score for Pediatrics (CRISP) [6].

### 3.2 Interventional Procedure

A percutaneous transfemoral antegrade approach was performed in all patients. Omnilink Elite stents were used in 8 patients, while Herculink, Xience Prime and Promus Premiere were used in one patient. The average stent diameter was 8.50 (2.03) mm with an average length of 24.45 (7.94) mm. Before implanting the stent, all patients received additional pre-dilation to expand the stent diameter and enhance its adhesion. The details of the interventional procedure are summarized in Table **2**. Patient 9 had an additional PDA stent using Abbott Vascular Omnilink Elite 10.0 mm x 19 mm as, while patient 11 had Promus Premiere 3.5 mm x 16 mm.

### 3.3 Acute Results and Follow-up

**Fig. (1):**
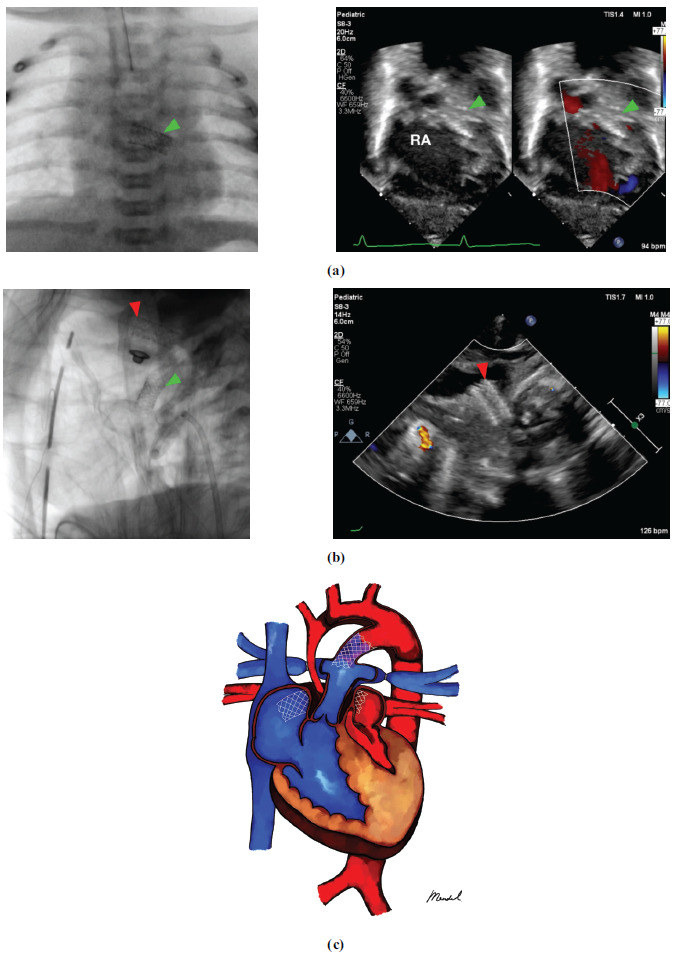
Concurrent patent ductus arteriosus (PDA) and inter-atrial septum (IAS) stenting in a patient with hypoplastic left heart syndrome (HLHS). (**a**) IAS stenting was conducted in this patient, but due to desaturation (showed by the *green arrowhead*), (**b**) PDA stenting was also performed (showed by *red arrowhead*) (**c**) Illustration. Notes: RA *right atrium*.

The placement of atrial stents without constriction was successful in 90.90% of the procedures, encompassing 10 patients. During the procedure for patient 8, the stent shifted into the right atrium while still positioned over the guidewire, resulting in the procedure's failure. Subsequently, the displaced stent was meticulously compressed and affixed to the right atrial free wall using sutures. In patient 9, following successful stent deployment, the patient developed severe bradycardia (HR 42), and CPR was attempted; nonetheless, the patient died on the operation table. Patients 9 and 11 underwent both inter-atrial septum stenting and patent ductus arteriosus stenting (Fig. **1**). Patient 11 died in the intensive care unit as a result of heart failure. Due to the inadequate healthcare system and accessibility in our country, most of these individuals presented late and had no previous heart surgery or cardiac intervention [7, 8]. Patient 7 had a successful IAS stent placement, however, excessive diuretics combined with recurrent diarrhea caused hypovolemic shock and death in the ward. Individual intra- and post-procedural complications data can be seen in Table **3**.

All patients encountering complications exhibited a CRISP risk of no less than 14.4% for a significant adverse event, while those who passed away had a CRISP risk of 36%. Unfortunately, because the number of subjects was so small, this number may not accurately reflect the issue. During the last follow-up echocardiography in all of the patients, no stent thrombosis was observed.

## 4 DISCUSSION

### 4.1 Outcomes of Inter-atrial Septum Stenting

During the last three decades, it has been objectively demonstrated that inter-atrial septum stenting temporarily improves patients' conditions, eliminates congestive heart failure and syncopes, and serves as a “bridge” to definite treatment in patients with duct-dependent physiology [1, 2, 5].

In a somewhat large case series, 18-39% of patients who survived the hospital period needed re-intervention due to spontaneous closure or restriction of interatrial communication after balloon atrial septostomy or the use of fenestrated occluders [9, 10]. Even though it has a more stable position, fenestrated devices have potential drawbacks, such as the technical inability to expand interatrial connection if clinically necessary.

Bar-Cohen [10] *et al*. (2005) conducted a study assessing seven neonatal patients with single ventricle physiology who received atrial septal stents (average weight 3.1 kg, range 2.4-4.3 kg), comparing them to eight neonates undergoing transseptal puncture and balloon dilation. There were no complications in the stented group *vs* 2 cases in the non-stented group. Danon [11] *et al*. (2005) investigated the long-term outcomes of 13 patients (median age 2 years, range 1 day to 20 years), where only one mortality was reported after the procedure. The remaining 12 stents were either removed during future surgery or remained patent and in place during subsequent echocardiography.

Mainzer *et al*. [12] (2018) successfully placed stents in 27 of 29 patients, with eight complications. Most problems (right atrial perforation, blood loss, femoral vein damage, pneumothorax, VF, and SVT) were easily managed. Three patients had their stents displaced. Four patients died as a result of clinical instability rather than severe consequences from the procedure itself. Complications and fatalities were predominantly observed in patients weighing 3 kg or less during the procedure, with a complication rate of 71% and a mortality rate of 43%. There were 14% procedural complications, such as stent displacement and severe bradycardia (Table **3**) and 5% death in the group of patients weighing more than 3 kg.

Putting a stent precisely in the atrial septum can be difficult, and echocardiography should be used to help, along with fluoroscopy and angiography if necessary [13-15]. In atrial septal stenting, echocardiography guidance plays a pivotal role in ensuring proper alignment of the middle segment of the stent with the atrial septum. The atrial septum would be more expansile in small body weight babies, requiring a bigger stent to assure stability in those groups. The dimensions of the stent vary across studies. It's crucial to have a stent length that facilitates smooth insertion across the septum, considering the small landing area and the angle relative to the catheter and guidewire upon release, without impacting the posterior wall of the left atrium. The diameter is selected to maintain the integrity of the atrioventricular valve plane while ensuring satisfactory patency [16, 17].

The atrial septum becomes thicker and more muscular after 6 weeks (>180 days). Because of this anatomical factor, it is extremely important to consider radial force while selecting a stent. With coronary stents, the lowest permissible collapse pressure is 300 mmHg. Due to its low radial strength of 1,096 mmHg, the cobalt renal stent is preferred for establishing a reliable and patent connection between the atrial chambers. Following interatrial stenting, the atrial septal defect was estimated to be approximately 5 mm in dimension. The labeled size of the stent always exceeds its primary orifice or inner diameter [18-20].

### 4.2 Additional PDA Stenting in Conjunction with IAS Stenting

In duct-dependent physiology, PDA stenting combined with atrial septal stenting as a bridge is a good strategy because it theoretically combines the advantages of PDA to the regressing left ventricle with adequate interatrial mixing for effective systemic and pulmonary blood flows. Furthermore, this method can be conducted in remote places and may reduce some of the hazards associated with the surgical approach (mortality, morbidity, duration of hospital stay, and economic effect) [19].

Regrettably, patient 11, who underwent PDA stent and IAS stent simultaneously, died while in the intensive care unit. We suspected that this condition was triggered by pulmonary reperfusion injury. The pathophysiology of PRI includes both ischemia and reperfusion injury, which results in oxidative stress from the formation of reactive oxygen species (ROS), endothelial dysfunction, and a cytokine storm that can cause numerous organ dysfunction [19-21]. Extracorporeal membrane oxygenation (ECMO) was available at our facility, but it was never employed in conjunction with extracorporeal cardiopulmonary resuscitation (ECPR) because there are so few studies on its use in single ventricle physiology. The patient's normothermia was preserved during the treatment, and neither cardiopulmonary bypass (CPB) nor ECMO were utilized.

### 4.3 Severe Bradycardia after IAS Stenting

Patients undergoing percutaneous coronary intervention frequently experience vasovagal reflex syndrome. However, there has been no case of vasovagal reflex syndrome following IAS stenting. Vasovagal reflex syndrome is defined as a complicated hemodynamic reaction with bradycardia, severe hypotension, and loss of consciousness. Additionally, thrombus occurs promptly at the stent position if the vasovagal reaction is not reversed within 10 minutes [22].

Previous research has linked various conditions to the vasovagal reaction, including sheath removal, reduced venous return, and cavity viscera dilation. Transitory disturbances followed by spontaneous recovery are common with normal heart rhythm and blood pressure. Nonetheless, it can occasionally be a serious, even deadly, case. A disproportionately mature parasympathetic system in the duct-dependent circulation may predispose to sudden severe bradycardia. According to our findings, 7.69% of patients getting IAS stenting experienced vasovagal response and died. It is generally recognized that a drop in blood pressure is caused by a significant decrease in cardiac output and vascular resistance, whereas a change in heart rhythm is caused by vagus nerve stimulation [22-24].

## 5 LIMITATION

There are several limitations to our study. To begin with, this is a retrospective study with no control group or randomization, which could help to reduce bias. Only a few research articles on inter-atrial septum stenting have been published, and they are primarily sporadic case reports or small case series. Additionally, the proportion of patients in the compared groups with varying levels of risk is unequal. Finally, the duration of follow-up is rather brief and varies greatly between groups.

## CONCLUSION

Inter-atrial septum stenting is a rare procedure, despite having a high success rate in patients who require stable septal patency. In addition, more studies are required in individuals with duct-dependent physiology PDA stent performed concurrently with IAS stent.

## AUTHORS’ CONTRIBUTIONS

Radityo Prakoso conceived the original idea of the manuscript, and all authors discussed and agreed with the idea of the paper. Radityo Prakoso, Yovi Kurniawati, Sisca Natalia Siagian, Aditya Agita Sembiring, Damba Dwisepto Aulia Sakti and Brian Mendel contributed to collecting data and writing the main text of the paper. The manuscript was proofread and accepted by all authors.

## Figures and Tables

**Table 1:** Baseline characteristics.

Patients	11
Female/Male	4/7
Age at implantation [Median(range)]	150 days (11 days - 703 days)
Birth weight [Median(range)]	2.8 (2.5-3.2) kg
Weight at implantation [Median(range)]	3.9 (2.8-9) kg
**Physiology**	
Hypoplastic left heart syndrome	9 (81.82%)
Transposition of great arteries	3 (27.27%)
Pulmonary atresia/VSD	2 (18.18%)
**Procedural Risk for Serious Adverse Events**	
CRISP 5 (36.8% for SAE)	5 (45.45%)
CRISP 4 (14.4% for SAE)	6 (54.54%)
CRISP 3 (6.2% for SAE)	0 (0%)
CRISP 2 (2.6% for SAE)	0 (0%)
CRISP 1 (1.0% for SAE)	0 (0%)

**Abbreviations:** VSD ventricular septal defect, CRISP catheterization risk score for pediatrics, SAE serious adverse events.

**Table 2:** Interventional procedure.

**Type of Procedure**	
IAS stent	11 (100%)
Additional PDA stent	2 (18.18%)
Additional surgical procedure	2 (18.18%)
**Approach**	
Antegrade transfemoral	11 (100%)
**Stent Size - Mean (SD)**	
Diameter	8.50 (2.03) mm
Length	24.45 (7.94) mm
Pre-procedural saturation - [median(range)]	65 (25-89) %
Post-procedural saturation - [median(range)]	81 (72-94) %
Procedural success rate	10/11 (90.90%)

**Abbreviations:** IAS inter-atrial septum, PDA patent ductus arteriosus, SD standard deviation.

**Table 3:** Individual patient characteristic.

Patient	Gender	Age (Days)	Weight (kg)	CRISP Score	Diagnosis	Stent	Stent Implantation	Pre-procedural Saturation (%)	Post-procedural Saturation (%)	Stent Condition During Last Follow-up Echocardiography	Perioperative/ 30-day Mortality	Intra-procedural Complications	Post-procedural Complications
1	Male	182	3.9	4	Mitral atresia, large muscular-inlet TGA-VSD, bilateral SVC post tight PA banding and PDA ligation	Omnilink elite 10x19 mm	Success	77	85	Patent	No	None	None
2	Female	703	9	4	Mitral atresia, DORV, muscular-outlet VSD, hypoplastic LV, bilateral SVC post PA banding and PDA ligation	Omnilink elite 9x29 mm	Success	84	94	Patent, stent removed after BCPS surgery	No	None	None
3	Male	61	4.8	5	Mitral atresia, DORV, muscular VSD, hypoplastic LV, restrictive PFO	Omnilink elite 10x29 mm	Success	52	81	Patent	Yes	None	Death due to sepsis and failure at day 14
4	Male	58	3.7	4	Mitral atresia, restrictive ASD	Omnilink elite 10x29 mm	Success	72	85	Patent	Yes	None	Death due to ventilator-associated pneumonia at day 22
5	Male	11	2.8	4	HLHS	Herculink elite 6x12 mm	Success	40	75	Patent	Yes	None	Death one hour surgical-related post re-tightening PA banding
6	Female	666	7.2	4	Mitral atresia, DORV, muscular VSD, hypoplastic LV, restrictive PFO	Omnilink elite 10x29 mm	Success	89	92	Patent	No	None	None
7	Male	150	4.6	5	HLHS, stretched PFO L-R shunt, small muscular VSD, large PDA R-L shunt	Omnilink elite 9x29 mm	Success	65	80	Patent	Yes	None	Death due to acute gastroenteritis and metabolic acidosis at day 4
8	Male	295	3.1	5	TGA-IVS, PDA, PFO	Omnilink elite 8x19 mm	Failed	25	74	N/A	Yes	Dislodged stent to right atrium	N/A
9	Female	35	3.1	5	HLHS variant-hypoplastic mitral, large PDA, PFO, bilateral SVC	Xience Prime 9x19 mm	Success	48	72	Patent	Yes	Severe bradycardia, death on table	N/A
10	Male	693	9	4	Mitral atresia, PA-VSD, hypoplastic LV, restrictive secundum ASD, right aortic arch	Omnilink elite 9x39 mm	Success	83	83	Patent	Yes	None	Death due to pneumonia and right lung atelectasis at day 26
11	Female	60	3.3	5	PA-VSD, mitral atresia, restrictive PFO, PDA	Promus premiere 3.5x16 mm	Success	52	78	Patent	Yes	None	Death due to failure at day 5

**Abbreviations:** BCPS bicaval pulmonary shunt, CRISP catheterization risk score for pediatrics, TGA-VSD transposition of the great arteries-ventricular septal defect, SVC superior vena cava, PA pulmonary artery, PDA patent ductus arteriosus, DORV double outlet right ventricle, LV left ventricle, PFO patent foramen ovale, PA-IVS pulmonary atresia-intact ventricular septum, RV right ventricle, HLHS hypoplastic left heart syndrome.

## Data Availability

The data and supportive information are available within the article.

## LIST OF ABBREVIATIONS

CHD: Congenital Heart Disease
EMR: Electronic Medical Record
IAS: Inter-atrial Septum
mBTTS: Modified Blalock-Thomas-Taussig Shunt
PDA: Patent Ductus Arteriosus

## STANDARDS OF REPORTING

STROBE guidelines were followed.
